# Methylenetetrahydrofolate Reductase A1298C Polymorphism and Major Depressive Disorder

**DOI:** 10.7759/cureus.1734

**Published:** 2017-10-01

**Authors:** Kevin Cho, Zubair M Amin, Jie An, Kerry Anne Rambaran, Tyler B Johnson, Saeed K Alzghari

**Affiliations:** 1 Reference Health Laboratories, Gulfstream Diagnostics; 2 Thomas J. Long School of Pharmacy & Health Sciences, University of the Pacific; 3 Gulfstream Genomics, Gulfstream Diagnostics; 4 Department of Clinical Sciences, Keck Graduate Institute

**Keywords:** depression, homocysteine, single nucleotide polymorphisms, methylenetetrahydrofolate reductase, polymorphism, mthfr, major depressive disorder, a1298c

## Abstract

Major depressive disorder (MDD) is a disorder that carries significant psychosocial and economic implications. Research efforts have focused on identifying biomarkers that can aid in the prediction, diagnosis, and efficacious treatment of MDD. Most of this focus has been placed on a polymorphism of the methylenetetrahydrofolate reductase (*MTHFR*) gene, C677T. *MTHFR* C677T is screened during MDD diagnosis in many protocols. However, *MTHFR* C667T poses conflicting data in various ethnic groups and geographic populations calling into question its utility. Another polymorphism, *MTHFR *A1298C, has often taken the back-seat to *MTHFR *C677T in respect to research focus. *MTHFR *A1298C is implicated in irregular homocysteine metabolism and aberrant folate cycles and, through this, it may play a role as either a driver in the development of MDD or as a predictive or diagnostic marker, possibly in combination with C677T. The number of studies evaluating *MTHFR* A1298C and the power of those studies is lacking and thus larger studies are required to confirm the association between this polymorphism and MDD.

## Introduction and background

Major depressive disorder (MDD) or clinical depression, is a mental disorder characterized by a persistent feeling of hopelessness or despair and a general loss of interest in daily activities. Depression can have a significant impact on a person’s well-being, often negatively affecting one’s daily behavior, sleeping habits, self-esteem, or energy levels [1]. In 2015, nearly 16 million adults in the United States (U.S.) had experienced MDD within the previous year, and this number accounts for approximately 7% of all adults in the U.S. [2]. Given the prevalence and socioeconomic implications of depression, greater research emphasis is being placed on the enigmatic and complex biochemical reactions that underlie depression. Some researchers, like a group at Harvard, have sought to understand links between the slow production of new neurons and periods of depression. Interestingly, these conclusions might help to explain why there is a delayed reaction of several weeks when some individuals undergo treatment with antidepressants [3]. Other groups have begun to explore connections between genetic polymorphisms in genes such as methylenetetrahydrofolate reductase (*MTHFR*) and an individual’s propensity towards major depression. The *MTHFR* gene encodes the enzyme called methylenetetrahydrofolate reductase (MTHFR). This enzyme plays an important role in the folate-cycle through the conversion of 5, 10-methylenetetrahydrofolate to 5-methyltetrahydrofolate (5-MTHF). 5-MTHF will then donate a methyl group in the conversion of homocysteine (HCY) to methionine [4]. A lack of MTHFR or expression reduced-function variant of the enzyme will lead to decreased levels of 5-methyltetrahydrofolate, thus resulting in high levels of HCY. Both irregular folate metabolism and high levels of HCY have shown to be correlated with MDD and may be indicators of polymorphisms in the *MTHFR *gene [5-7]. Furthermore, there is evidence that has suggested both the *MTHFR *C677T (dbSNP: rs1801133) and A1298C (dbSNP: rs1801131) single nucleotide polymorphisms (SNPs) are correlated to MDD and its treatment [7-9]. While much research focus has been placed on *MTHFR* C677T as the primary polymorphism of concern, recent mounting evidence also implicates A1298C as another important polymorphism of the *MTHFR *gene. In the following review, we seek to highlight evidence of the single nucleotide polymorphism, *MTHFR* A1298C, in the context of MDD.

## Review

The aim of this review was to assess the *MTHFR* A1298C polymorphism and its relationship to MDD. A literature search for articles in PubMed, ISI Web of Knowledge, and Scopus was performed. The terms for the search strategy were “MTHFR,” “A1298C,” and “depression.” We utilized the following criteria for article selection: 1) articles written in English; 2) must be full-text articles; and 3) must be either a randomized controlled trial, prospective trial, retrospective analysis, case series, or case report. We examined the reference lists of each article for additional resources that fit the selection criteria. We initially identified 15 articles. We excluded an article related to bipolar disorder, an article that failed to isolate depression patients’ MTHFR status, an article related to ophthalmology, five meta-analyses, and one review article. In total, six relevant articles were identified for this review and we summarize our findings in Table 1 [6-11].

**Table 1:** PICO analysis of current MTHFR A1298C major depressive disorder studies *Statistically significant (p ≤ 0.05) Abbreviations: cDNA- complementary deoxyribonucleic acid; CES-D- Center for Epidemiologic Studies Depression Scale; COMT- catechol-O-methyl transferase; DSM- Diagnostic and Statistical Manual of Mental Disorders; MDD- major depressive disorder; HCY- homocysteine; htSNP- haplotype tagging single nucleotide polymorphism; MADRS- Montgomery-Åsberg Depression Rating Scale; MTHFR- methylenetetrahydrofolate reductase; PHQ- Patient Health Questionnaire; RNA- ribonucleic acid; SNP- single nucleotide polymorphism; SSRI- selective serotonin reuptake inhibitor Note: All major depressive disorder patients were diagnosed by DSM-IV or DSM-V criteria

Author (Year)	Population	Intervention	Control	Outcome
Reif et al. (2005) [8]	Female inpatients with acute psychiatric disorders (n=120) Bipolar disorder and MDD inpatients in Germany (n=136)	Genotyping SNPs: C677T & A1298C Determination of HCY, vitamin B12, & folate levels	Healthy blood donors from the same region with low chance of MDD (n=284)	Severity associated with high HCY level Elevated HCY associated with elderly_­_* A1298C CC genotype associated with MDD and bipolar disorder*
Evinova et al. (2012) [9]	Patients with MDD (n=134)	Genotyping SNPs associated with MDD: G196A, C677T, & A1298C	Healthy blood donors without MDD (n=143)	A1298C: Allele C frequency associated with 39.2% MDD and 29.7% control; CC in men at more risk for MDD*
Bousman et al. (2013) [10]	Patients enrolled in Diamond Study with MDD (n=147)	Genotyping SNPs: C677T, A1298C, and 7 other gene-specific htSNPs DSM, CES-D, & PHQ severity assessment at various time intervals (t = 0, 24, 36, 48, & 60 months)	Baseline measures of the patients (t=0) 7 htSNPs	No association between A1298C and MDD prognosis
Jamerson et al. (2013) [11]	Elderly patients (age >60) with MDD who are prescribed with SSRI (n=104)	MADRS severity assessment at every 3 months for remission determination Genotyping 15 SNPs from 10 genes from folate metabolism Folate intake estimated via Block 1998 Questionnaire	SSRI treatments Folate intake	A1298C AC genotype associated with 2.5x likelihood with SSRI treatment*
Nielsen et al. (2015) [6]	Patients with MDD who were resistant (n=389) and responsive (n=224) to antidepressants	Genotyping MTHFR and COMT polymorphism: C677T, A1298C, & Val158Met cDNA synthesis from fibroblasts RNA	Healthy volunteers without MDD (n=463)	A1298C CC genotype more frequent in female MDD patients than controls* A1298C significant interaction between gender and allele A carriers* A1298C CC genotype associated with lower age of onset
Mech et al. (2016) [7]	Patients with positive C677T and A1298C SNPs (n=159)	Administration of EnLyte® (reduced B vitamins) MADRS severity at various time interval (t = 0, 2, & 8 weeks)	Positive C677T and A1298C SNPs Administration of placebo (n=123) MADRS (t=0)	Reduction in homocysteine levels at week 8 in 82.4% of active treatment patients* Full remission achieved by week 8 in 42% of active treatment patients*

A1298C and risk of depression

A transversion at nucleotide 1298 in exon 7 on the *MTHFR* gene leads to a 60% reduction in enzyme activity compared to the wild-type enzyme [12]. While the C677T transition does lead to greater reduction of the enzymatic activity of MTHFR than that of A1298C, the current findings on C677T alone are conflicting and limit association between the genotype and its implication in MDD [13-16]. A few recent studies examined the association of both C677T and A1298C over the same population that may alleviate the aforementioned conflicts. By genotyping 136 female patients and 284 controls in a study by Reif, et al., A1298C was determined to be significantly associated with both MDD and bipolar disorder whereas C677T failed to find a similar association [8]. Another study by Evinova, et al. enrolling 143 patients and 134 controls showed that C allelic frequency and CC genotype male carriers were significantly associated with MDD incidence [9]. These two studies by Reif, et al. and Evinova, et al. delve into genetic variations in the Caucasian population, specifically German and Slovak ancestries, respectively, in which the prevalence in polymorphisms of the CC genotype in A1298C (8.54%) is lower than the TT genotype in C677T (12.5%) [11]. Additionally, the TT genotype of C677T appears to have modest effect on the risk of MDD [10, 13, 17]. A recent study by Nielsen and co-workers on A1298C failed to find the same association between A1298C and MDD despite assessing a large population of 613 patients and 463 controls [6]. The study, however, additionally noted that the strong association was found only in female patients and controls with higher statistical power than the previous studies. Based on these findings, A1298C has the potential to be an alternative or complementary, gender-specific indicator in diagnosing MDD in the Caucasian population. However, additional studies with a larger population size are needed to validate this.

Other studies investigated the association between the A1298C genotypes and MDD prognosis. In one small study by Bousman et al. with variable treatment modality, A1298C did not show any association between its genotypes and MDD prognosis, whereas C677T CC genotype had greater severity scores and indicated poor remission over 60 months [10]. However, Jamerson et al. found that the A1298C AC genotype is significantly more likely to be in remission as compared with the A1298C AA genotype when subjected to selective serotonin reuptake inhibitor (SSRI) treatments [11]. While the *MTHFR* gene has the potential to predict MDD prognosis by assessing both SNPs, their strong correlation yet remains to be elucidated. Further, these studies suggest that the prognosis may not depend solely on reduced enzyme activity. Alternatively, they suggest C677T and A1298C may affect HCY level to varying degree.

A1298C, diet, and homocysteinemia

MTHFR regulates the conversion of HCY to methionine through catalyzing the formation of 5-MTHF. Reduced enzyme activity through function-reducing polymorphisms results in the impairment of HCY metabolism and the folate cycle. Impairment of these processes results in the inadequate synthesis of serotonin and myelin as well as aberrations in other neural and vascular pathways [18]. Only a few studies have investigated the link between A1298C and HCY metabolism, yielding conflicting results [19-23].

In the study by Jamerson, et al., nutritional folate intake in a geriatric population was monitored along with genetic sampling for genes associated with folate metabolism [11]. The study demonstrated a slight increase in mean total folate level in the non-remitting population but no robust association. Similarly, serum folate level was normal in all cases, although 16 out of 24 patients had elevated HCY in the study performed by Reif, et al. [8]. Based on these findings, dietary folate intake does not appear to affect the level of HCY or predisposition of depression. However, elevated HCY levels were still observed in the same population that may suggest dietary folate conversion to the co-substrate is impaired. In a recent study by Mech and Farah, this idea was investigated by administering the metabolized vitamins and micronutrients in a randomized double-blind study [7]. Mech and Farah showed that 82.4% of 159 patients with a positive C677T or A1298C polymorphism demonstrated a reduction in HCY levels when treated with reduced vitamin B while 123 controls demonstrated a slight increase in HCY from baseline, which was statistically significant [7]. Because their study used a population that included either SNP, HCY elevation due to A1298C could not be accurately predicted. Furthermore, it was found in previous studies that A1298C has a significant effect on enzyme activity, but was not strongly associated with increased HCY concentrations [12].

In addition, Mech and Farah showed that 42% of patients with either an *MTHFR *A1298C or C677T polymorphism achieved full remission by week eight compared to the control population that was given placebo, while other studies that included elderly patients agree with similar associations between HCY level and MDD by noting that HCY level is strongly associated with age [7]. Despite the controlled folate intake, vascular dysfunction manifests into psychiatric symptoms which place the elderly population more at risk for poorer outcomes related to diet and genetic differences in folate genes [8, 11, 24].

Limitations

The primary limitation to A1298C analysis is the lack of large studies of high statistical power. Furthermore, a dearth of randomized controlled trials on this topic exist and most of the data presented are from observational studies. Although the aforementioned trials may not have been powered, many of the A1298C studies showed statistical significance associated with depression risk and homocysteinemia that are worth evaluating in larger trials.

Future directions

Future A1298C studies should examine large populations in various ethnicities and geographic locations. Diagnostic and Statistical Manual of Mental Disorders, Fifth Edition (DSM-V) and the anticipated International Classification of Diseases (ICD)-11 should continue to give researchers proper guidance in selecting such populations for study. Due to subjectivity, a combination of rating scales should be administered to assess the remission of MDD such as the Montgomery–Åsberg Depression Rating Scale (MADRS), the Patient Health Questionnaire-9 (PHQ-9), the Center for Epidemiologic Studies Depression Scale (CES-D), or the Composite International Diagnostic Interview (CIDI). Additionally, more randomized studies should focus on solely examining the influence of A1298C instead of both A1298C and C677T to better isolate the influence of A1298C. Finally, according to a retrospective cross-sectional study evaluating Medicare claims, a total of 182,358 *MTHFR* tests were billed in 2013 [25]; thus, a consideration of A1298C as part of a larger decision algorithm for clinicians currently utilizing the *MTHFR *gene in practice should be incorporated as an option for patients with MDD [26].

## Conclusions

Based on current studies, the *MTHFR* A1298C polymorphism has the potential to be an alternative or complementary gender-specific indicator in MDD diagnosis, but requires further study. The role of A1298C either by itself or in combination with *MTHFR* C677T in MDD prognosis must be further elucidated. Additionally, the comparative measures of the likelihood of MDD between the two polymorphisms must also be examined.
